# Analysis of macaque *BTN3A* genes and transcripts in the extended MHC: conserved orthologs of human γδ T cell modulators

**DOI:** 10.1007/s00251-019-01126-9

**Published:** 2019-08-05

**Authors:** Nanine de Groot, Rens Groen, Vaneesha Orie, Jesse Bruijnesteijn, Natasja G. de Groot, Gaby G. M. Doxiadis, Ronald E. Bontrop

**Affiliations:** 1 Department of Comparative Genetics and Refinement, Biomedical Primate Research Centre, Lange Kleiweg 161, 2288 GJ Rijswijk, The Netherlands; 2 Department of Theoretical Biology and Bioinformatics, Utrecht University, Utrecht, The Netherlands

**Keywords:** BTN3A, MHC, Macaques, Gamma-delta T cells, Alternative splicing

## Abstract

Butyrophilins (BTN), specifically BTN3A, play a central role in the modulation of γδ T cells, which are mainly present in gut and mucosal tissues. BTN3A1 is known, for example, to activate Vγ9Vδ2 T cells by means of a phosphoantigen interaction. In the extended HLA region, three genes are located, designated *BTN3A1*, *BTN3A2* and *BTN3A3*, which were also defined in rhesus macaques. In contrast to humans, rhesus monkeys have an additional gene, *BTN3A3Like*, which has the features of a pseudogene. cDNA analysis of 32 Indian rhesus and 16 cynomolgus macaques originating from multiple-generation families revealed that all three genes are oligomorphic, and the deduced amino acids display limited variation. The macaque *BTN3A* alleles segregated together with MHC alleles, proving their location in the extended (Major Histocompatibility Complex) MHC. BTN3A nearly full-length transcripts of macaques and humans cluster tightly together in the phylogenetic tree, suggesting that the genes represent true orthologs of each other. Despite the limited level of polymorphism, 15 *Mamu*- and 14 *Mafa-BTN3A* haplotypes were defined, and, as in humans, all three *BTN3A* genes are transcribed in PBMCs and colon tissues. In addition to regular full-length transcripts, a high number of various alternative splicing (AS) products were observed for all *BTN3A* alleles, which may result in different isoforms. The comparable function of certain subsets of γδ T cells in human and non-human primates in concert with high levels of sequence conservation observed for the BTN3A transcripts presents the opportunity to study these not yet well understood molecules in macaques as a model species.

**Electronic supplementary material:**

The online version of this article (10.1007/s00251-019-01126-9) contains supplementary material, which is available to authorized users.

## Introduction

T cells are an essential component of the adaptive immune system and use cell surface bound receptors (TCRs) to recognize ligands that may signal the presence of intracellular infections or cellular stress. In addition to T cells that express a T cell receptor composed of a heterodimer of an α and a β chain, another T cell lineage expresses a different set of receptors consisting of a γ and a δ chain instead. Although much less abundant than αβ T cells in peripheral blood, these γδ T cells are highly plentiful in tissues such as skin, the intestines and the reproductive tract (Adams et al. 2015). Moreover, they represent an important effector subset of innate-like T lymphocytes (Rhodes et al. 2016). In contrast to TCRs of the αβ lineage, however, which recognize antigenic peptides in the context of classical or non-classical MHC class I (MHC-I) molecules, some γδ-T cells recognize lipid molecules presented by MHC-like molecules such as CD1d and CD1c. Additionally, there are others, which can directly detect antigens, sometimes even without presentation via a canonical, antigen-presenting MHC or MHC-like molecule (Bai et al. 2012; Luoma et al. 2014; Roy et al. 2014). The most thoroughly investigated subset represents the Vγ9Vδ2 T cells, which are the dominant population of γδ-T cells in human peripheral blood, with potent activity regarding certain microbial infections and cancers (Gober et al. 2003; Morita et al. 2007). Vγ9Vδ2 T cells respond specifically to pyrophosphates derived from microbial sources or malignant cells. These phosphoantigens (pAg) are recognized in a TCR-dependent manner, but in contrast to αβ-T cells, they apparently do not require MHC molecules for pAg-dependent activation. Recently, molecules belonging to the butyrophilin (BTN) protein family, specifically butyrophilin-3A (BTN3A), were identified as an essential component in the pAg activation pathway of Vγ9Vδ2 T cells (Abeler-Dorner et al. 2012; Afrache et al. 2012; Karunakaran and Herrmann 2014; Rhodes et al. 2016; Vavassori et al. 2013). BTN3A proteins are cell-surface receptors expressed on a broad variety of cell types, including immune cells and some malignant cells such as ovarian cancer (Arnett and Viney 2014). They belong to the Ig-like superfamily and are evolutionarily and structurally related to the cluster of B7 costimulatory molecules CD80 and CD86 (Abeler-Dorner et al. 2012; Afrache et al. 2012; Rhodes et al. 2016). They exhibit two extracellular domains, IgV and IgC (Williams and Barclay 1988), and a transmembrane part. In contrast to the B7 molecule, however, most BTN molecules also contain a cytosolic B30.2 (PRYSPRY) domain, which is separated from the transmembrane domain by several very short exons, which are called heptads (Rhodes et al. 2001). *BTN3A* genes belong to a multigene family, which maps in humans to the extended MHC class I region and comprises three genes: *BTN3A1*, *BTN3A2* and *BTN3A3* (Horton et al. 2004; Rhodes et al. 2001, 2016). *BTN3A1* and *BTN3A3* contain the intracellular B30.2 domain, which is absent in *BTN3A2*. Although the prominent role of *BTN3A1* in pAg-induced Vγ9Vδ2 T cell activation is well documented, the working mechanism is not yet fully understood, and the extracellular ligand for BTN3A1 molecules is still unknown. There is strong support for the intracellular sensing of pAg through the B30.2 domain of BTN3A1-mediating Vγ9Vδ2 T cell activation (Harly et al. 2012; Palakodeti et al. 2012; Peigne et al. 2017; Rhodes et al. 2015; Sandstrom et al. 2014). Additionally, crystallographic studies illustrated that BTN3A1 molecules may exist in two potential dimer conformations, and pAg-induced conformational changes of the intracellular domain of BTN3A1 might also involve dimer conversion (Gu et al. 2017, 2018; Palakodeti et al. 2012). While BTN3A1 is necessary for pAg-induced Vγ9Vδ2 T cell activation, this molecule alone may not be sufficient to conduct the activation process. Two cytoskeletal mediators have been identified, and named Periplakin and RhoB GTPase, which interact directly with BTN3A1, and may as such be involved in the activation process (Rhodes et al. 2015; Sebestyen et al. 2016). Additionally, recent work postulates that ABC transporters play a role in BTN3A-dependent γδ T cell activation by way of cytokine secretion (Rhodes et al. 2018). Furthermore, a co-expression and multimerization of BTN3A1 with its sister molecules BTN3A2 and BTN3A3 may be necessary for their function, comparable to a concerted action as documented for other BTN molecules (Di Marco Barros et al. 2016; Gu et al. 2018; Rhodes et al. 2015).

BTN3A proteins indeed play a role in T cell proliferation and cytokine production, which was manifested through the use of BTN3A-specific antibodies. One of the antibodies, 20.1, triggers Vγ9Vδ2 T cell activation, whereas a different antibody, 103.2, works as an antagonist and blocks pAg-induced T cell activation (Arnett and Viney 2014; Harly et al. 2012). The functional importance of BTN3A molecules was illustrated in a xenotransplantation model by the agonist antibody 20.1, which was able to enhance the Vγ9Vδ2 T cell-mediated killing of leukaemia cells (Benyamine et al. 2016). Moreover, the influence of BTN3A1 in stimulating anti-tumour effector Vγ9Vδ2 T cells in zolodronate-treated colorectal cancer cells was documented as well (Zocchi et al. 2017).

Mutations in *BTN* genes may influence disease outcome, since polymorphisms in *BTN3A2* are associated with an increased susceptibility to developing type I diabetes (Viken et al. 2009). *BTN3A* genes are highly conserved in evolution and have been defined not only in higher primates but also in some other placental mammals such as the alpaca (Karunakaran et al. 2014). The three human *BTN3A* genes are the result of two successful rounds of duplications during the radiation of primates (Afrache et al. 2017). All three *BTN3A* genes seem to be present in hominoids (humans and great apes) and Old World monkeys (OWM). The concerted evolution of these genes resulted in a strong homogenization of the IgV domain in hominoids, where the sequences of *BTN3A1* and *BTN3A3* are replaced by *BTN3A2*. This homogenization of the IgV domain appears to be absent in OWM (Afrache et al. 2017).

Despite the increasing interest in BTN3A as immune modulators of Vγ9Vδ2 T cells, nothing is known so far about the possible levels of polymorphism and their distribution in OWM species such as macaques. Therefore, large families of rhesus and cynomolgus macaques were analysed for their *BTN3A* genes by cloning and Sanger sequencing.

Already in the beginning of the century, alternative splicing (AS) was observed in addition to constitutive splicing by Sanger sequencing specifically for human BTN3A transcripts, indicating that AS occurs far more frequently in these genes than in others (Rhodes et al. 2001). With more recent NGS techniques, AS has been documented in various genes of the immune system as well, and the different splicing mechanisms have been extensively described (Bruijnestijn et al. 2018; Keren et al. 2010; Voorter et al. 2016). In the present study, various isoforms of macaque BTN3A transcripts that were generated by AS have been documented, and the most frequent splice events have been characterized in detail.

## Material and methods

### Animals

We analysed DNA samples of 32 rhesus macaques of Indian origin belonging to two families of four generations and 16 cynomolgus macaques of various origins including different Indonesian islands as well as the Indonesian continent, belonging to two families of two generations, respectively. These animals belong to outbred breeding colonies that are housed at the BPRC, the Netherlands, and had been extensively characterized beforehand for their MHC class I and II haplotypes (Doxiadis et al. 2013; de Groot et al. 2014).

Genomic DNA (gDNA) of exon 2 of one rhesus macaque family (family sire C68; no. 19) was analysed, followed by a cDNA analysis of all animals for exon 2 to exon 9 of the *BTN3A1* and *BTN3A3* genes, exon 2 to exon 7 of the *BTN3A2* gene and exon 2 of the pseudogene *BTN3ALlike.*

### Genomic DNA isolation and BTN3A exon 2 gDNA sequencing

gDNA of the selected animals was extracted from EDTA blood samples or from immortalized B cell lines using a standard salting out procedure. Exon 2 amplification was performed as described using adapted primers originally published by Rhodes and co-workers (Rhodes et al. 2001) (Suppl. Table 1). Amplicons were sequenced and analysed as described below.

### RNA isolation, cDNA preparation, cloning and sequencing

RNA was isolated from immortalized B cell lines or freshly prepared PBMCs (RNeasy kit, Qiagen) and was used for the synthesis of cDNA with the RevertAid First Strand cDNA synthesis Kit (Thermo scientific). *BTN3A1*, *BTN3A2* and *BTN3A3* sequences (exon 1 [partly] to exon 9) were gained by PCR on cDNA using newly designed primer sets for the specific amplification of all three BTN3As. The amplification of *BTN3A3Like* alleles was only successful by using a specific exon 2 5′primer and an exon 4 3′primer (Suppl. Table 1). Primers were synthesized by Invitrogen. PCR was performed in a 50-μl reaction containing 0.02 U/μl of Phusion High-Fidelity DNA polymerase enzyme with 0.3 μM of each primer, 250 μM dNTPs, 1× Phusion buffer HF, 3%DMSO (Invitrogen, Life Technologies) and 200 ng cDNA. The cycling parameters were a 2-min 98 °C initial denaturation step, followed by 30 cycles (35 cycles for BTN3A1) of a 30-s 98 °C denaturation step, a 30-s annealing step (54 °C for BTN3A1, 59 °C for BTN3A2 and 55 °C for BTN3A3) and a 90-s 72 °C extension step. A final extension step was performed at 72 °C for 7 min. PCR products were purified using a geneJet Gel Extraction Kit (Thermo Scientific™), and the purified amplicons were cloned into the pJET vector using the CloneJET PCR cloning kit, both according to the manufacturer’s guidelines (Thermo Scientific™). The cloned amplicons were then transformed in *Escherichia coli*-XL1-blue cells with the TransformAid Bacterial Transformation Kit (Thermo Scientific™). Per animal, a minimum of 32 clones was selected, and plasmid DNA was isolated using a standard mini-preparation procedure. The purified plasmid DNA was sequenced on an ABI 3500 genetic analyser (Applied Biosystems, Foster City, USA). The sequencing reaction was performed by 2 μM pJET primer, 1 μl BigDye terminator and 2 μl of 5× sequencing buffer in a total volume of 10 μl (Thermo Scientific™). The resulting sequences were analysed using the Sequence Navigator program (Applied Biosystems, Foster City, USA). BTN sequences were revised manually by applying the Lasergene 12 SeqMan Pro Sequence Alignment Editor. Allele definitions are based on at least two clones with identical sequences from different monkeys or on independent PCRs from one monkey. The sequences were named in accordance with the general nomenclature rules of the ImmunoPolymorphismDatabase (IPD-MHC database of nonhuman primates [NHP]) (de Groot et al. 2012) and sent to the NCBI database (Accession numbers: *BTN3A1*: LT992538-LT992545 and LT992494-LT992501; *BTN3A2*: LT992546-LT992555; *BTN3A3*: LT993304-LT993321 and *BTN3A1L*: LT993431-LT993437). The *BTN3A3* pseudogene sequence of the olive baboon was extracted from the NCBI database (Accession no. XM_009198763).

### Phylogenetic analysis of exon 2 and of exons 2–6 of BTN3A alleles

The evolutionary history of the *BTNA3* alleles was inferred using the neighbour-joining method and the optimal tree. The percentages of replicate trees in which the associated taxa clustered together in the bootstrap test (1000 replicates) are shown next to the branches. The tree is drawn to scale, with branch lengths in the same units as those of the evolutionary distances used to infer the phylogenetic tree. The evolutionary distances were computed using the maximum composite likelihood method and are in the units of the number of base substitutions per site. The analyses involved 37 (gDNA exon 2) and 56 (cDNA exon 2 to exon 6) sequences, respectively. Codon positions included were 1st + 2nd + 3rd + noncoding. All ambiguous positions were removed for each sequence pair. There was a total of 348 (exon 2) and 879 (exon 2 to exon 6) positions, respectively, in the final dataset. Evolutionary analyses were conducted in MEGA7.

### Alternative splicing

Alternative splice products observed for all BTN3A transcripts were categorized according to their frequency and splice mechanism (Suppl. Table 2). All observed splice events were substantiated by the prediction and scoring of the alternative and actual splice sites using three different in silico prediction tools. The selected tools were the Maximum Entropy Modeling Scan (MaxEntScan; MES) (Yeo and Burge 2004), the Position Weight Matrix (PWM) via SpliceView (Rogozin and Milanesi 1997) and the Human Splice Finder (HSF) (Desmet et al. 2009). These tools use different output value ranges, but the higher score always implies a better-predicted splice site. It should be noted that whether or not a splice event occurs, the scores do not provide a threshold, and the scores should only be used to compare related splice sites.

## Results

### Exon 2 analyses of Mamu-BTN3A genes

**Fig. 1:**
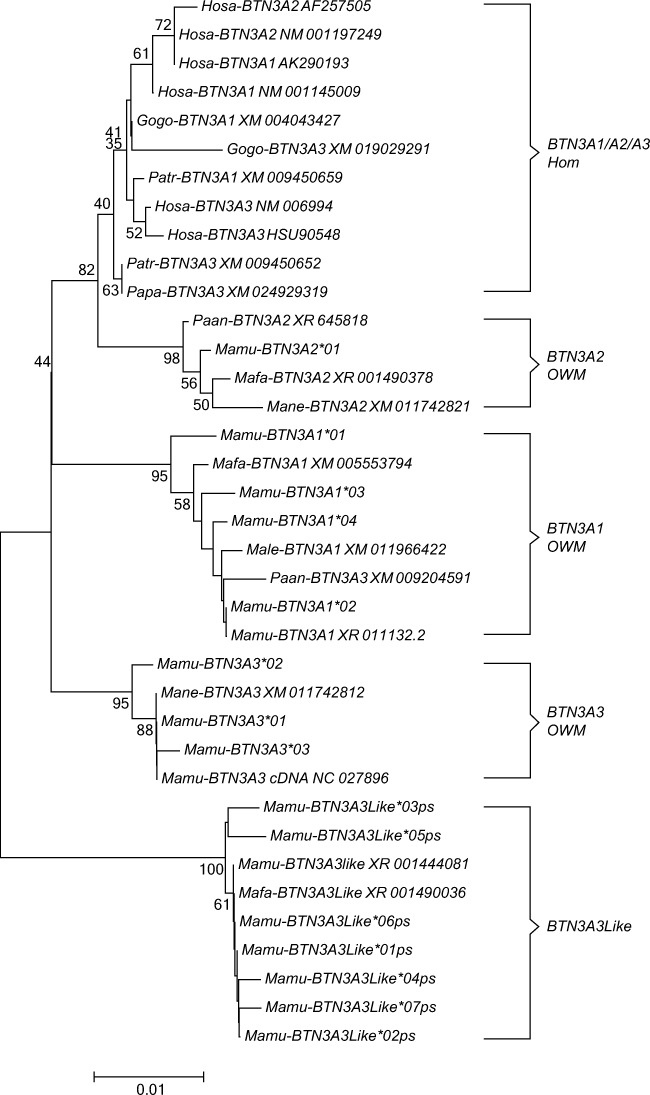
Phylogenetic tree of exon 2 of *BTN3A1*, *BTN3A2*, *BTN3A3* and *BTN3A3Like* of diverse primate species. Exon 2 sequences of the *BTN3A* genes and the *BTN3ALike* pseudogene of the rhesus macaque family EAW have been compared to *BTN3A* exon 2 sequences of humans (*Hosa*) and to various non-human primate species as deposited in the NCBI database. *Gogo Gorilla gorilla*, *Patr Pan troglodytes*, *Papa Pan paniscus*, *Paan Papio anubis*, *Mamu Macaca mulatta*, *Mafa Macaca fascicularis*, *Mane Macaca nemestrina*, *Male Mandrillus leucophaeus*

To scan for polymorphism of *BTN3A* genes in macaques, exon 2, encoding the IgV domain of the BTN3A genes of a four-generation, MHC-typed rhesus macaque family (family sire C68; #19), was sequenced at the genomic as well as the transcription level. All three *BTN3A* genes (*BTN3A1*, *3A2* and *3A3*) are present in the rhesus macaque. *BTN3A1* and *BTN3A3* are oligomorphic with four and three alleles, respectively, whereas in the studied cohort, *BTN3A2* did not show allelic variation in exon 2. Additionally, a fourth *BTN3A* gene was identified, seven alleles of which have been differentiated. Based on the similarity of this gene with *BTN3A3* sequences of other Old World Monkeys (OWM) extracted from the NCBI database, it was provisionally named *BTN3A3Like*. In rhesus and cynomolgus macaques, the sequences of this gene share a stop codon at triplet 65 of exon 2 near the end of this exon, leading to a truncated gene product, and therefore, this gene appears to be a pseudogene. Phylogenetic analyses of the rhesus macaque exon 2 alleles of the *BTN3A1*, *BTN3A2* and *BTN3A3* genes showed that they cluster together with alleles of the same gene of other OWM. In contrast, hominoid (Hom) exon 2 sequences of the three *BTN3A* genes fall apart from the gene clusters of OWM and do not branch in a gene-specific manner. Macaque *BTN3A3Like* alleles, however, constitute a separate cluster with a long branch length (Fig. 1), suggesting that this gene has undergone many mutations during evolution.

### Mamu- and Mafa-BTN3A allele discovery

**Fig. 2:**
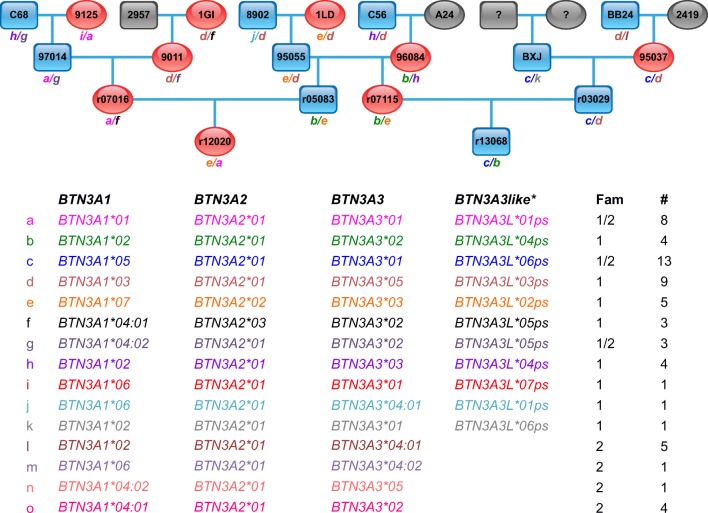
Pedigree of rhesus macaque family C68 and their deduced BTN3A haplotypes. Blue squares represent male and red ovals female animals. Grey squares/ovals indicate animals that have not been typed for BTN3A. Below the pedigree, the *BTN3A* haplotypes (designated a-o) are listed and the number of appearance (number sign) in family 1 (C68) and/or family 2 (EAW; Suppl. Fig. 1). For each animal, the inherited *BTN3A* haplotype is indicated in the pedigrees

**Fig. 3:**
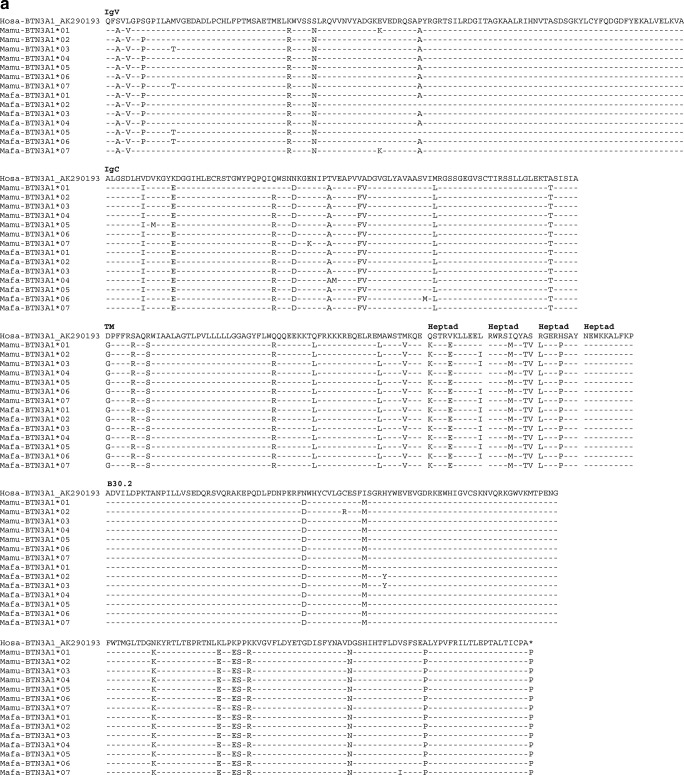

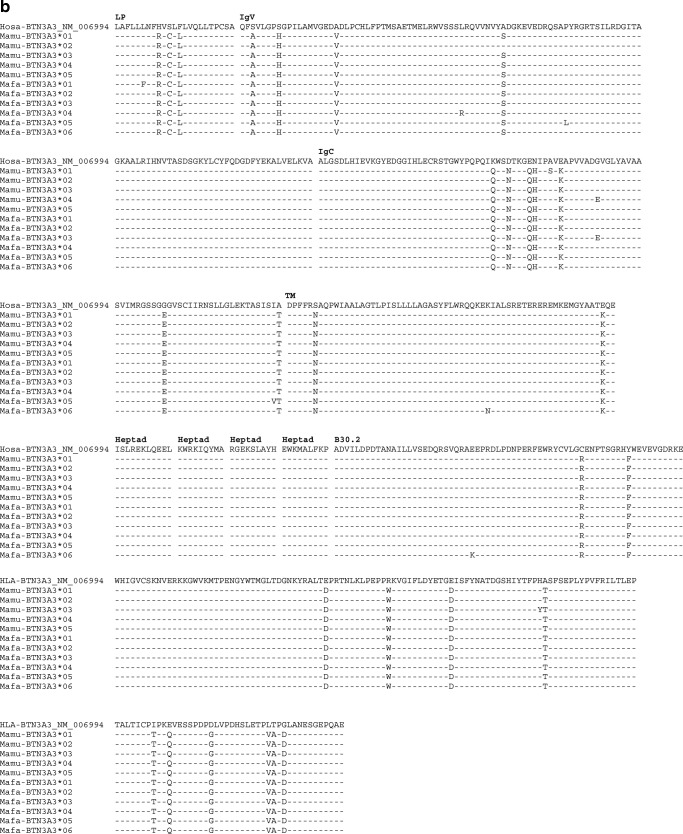

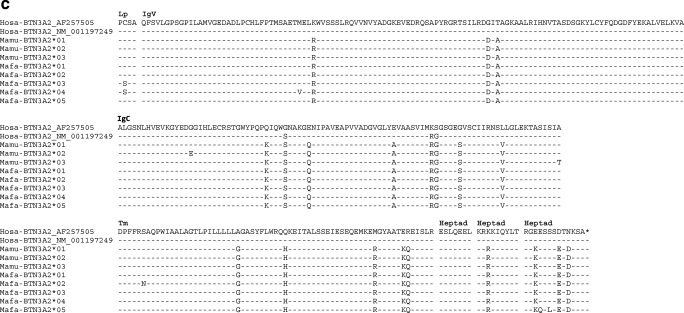
Alignment of BTN3A1-deduced amino acid sequences of rhesus (*Mamu*) and cynomolgus (*Mafa*) macaque BTN3A1 (**a**), BTN3A3 (**b**) and BTN3A2 (**c**); dash, identical amino acids; asterisk, not sequenced or stop codon

To determine the degree of allelic polymorphism in other exons and to assess whether the alleles defined in exon 2 represent *bonafide* transcripts, we analysed cDNA from exons 2–7 (partly) of *BTN3A2* and exons 2–9 of *BTN3A1/BTN3A3*. For *BTN3A3Like*, only exons 2 and 3 could be successfully amplified. However, like in exon 2, exon 3 of all *BTN3A3Like* alleles showed various stop codons, and therefore, no functional BTN products may be expected from these transcripts. This is why we did not use these sequences for further analyses as sequence or deduced amino acid comparisons. The analyses of the *BTN3A* transcripts of *BTN3A1*, *BTN3A2* and *BTN3A3* were performed for animals from the previously mentioned rhesus macaque family (sire C68, #19) (Fig. 2) and an additional four-generation rhesus macaque family (sire EAW; #13; Suppl. Fig. 1). The corresponding transcripts of all *BTN3A* alleles, which had been defined beforehand for exon 2, could be recovered. Most of them are shared between the two rhesus macaque families, and a total of eight *BTN3A1* and six *BTN3A3* alleles could be differentiated, which gave rise to seven and five different allotypes, respectively (Fig. 3a, b). With regard to polymorphic sites, only a few amino acid replacements were observed within the *BTN3A1* and *BTN3A3* genes of rhesus macaques, which suggest a high level of conservation. *BTN3A2* has a stop codon in exon 7, and therefore, as in humans, no B30.2 intracellular part is present at the transcript level. This molecule shows a very low degree of variation, with only three allotypes (Fig. 3c).

**Fig. 4:**
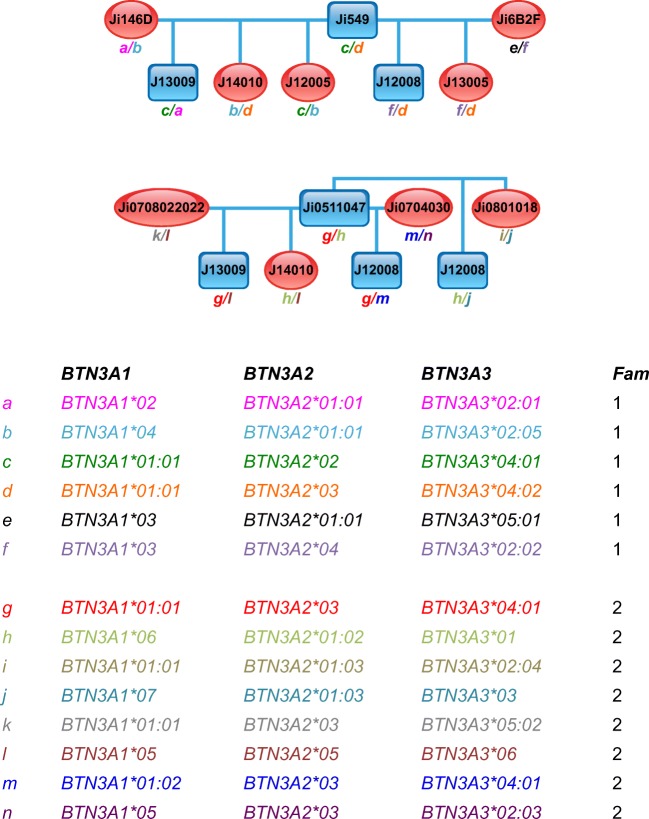
Pedigree of two cynomolgus macaque families and their deduced BTN3A haplotypes. Blue squares represent male, and red ovals represent female animals. Below the pedigree, the deduced BTN3A haplotypes (designated a–n) are listed, and the appearance in family 1 or 2 is indicated. For each animal, the inherited haplotype is indicated in the pedigrees

In addition, 16 cynomolgus macaques belonging to two families were analysed for their *BTN3A* polymorphism. Although fewer animals were tested than for rhesus macaques, an identical number of *BTN3A1* alleles have been recorded in both the cynomolgus and the rhesus macaque cohort, namely, eight of them, which also gave rise to seven different allotypes. Accordingly, cynomolgus macaques show more allelic variation for *BTN3A2* and *BTN3A3* with six and 12 alleles, which result in five and six different allotypes, respectively (Fig. 3a–c). In contrast to the rhesus macaque, in which both families share most of the *BTN3A* alleles, the two cynomolgus macaque families show mainly different alleles for *BTN3A1* and *BTN3A3* (Fig. 4).

**Fig. 5:**
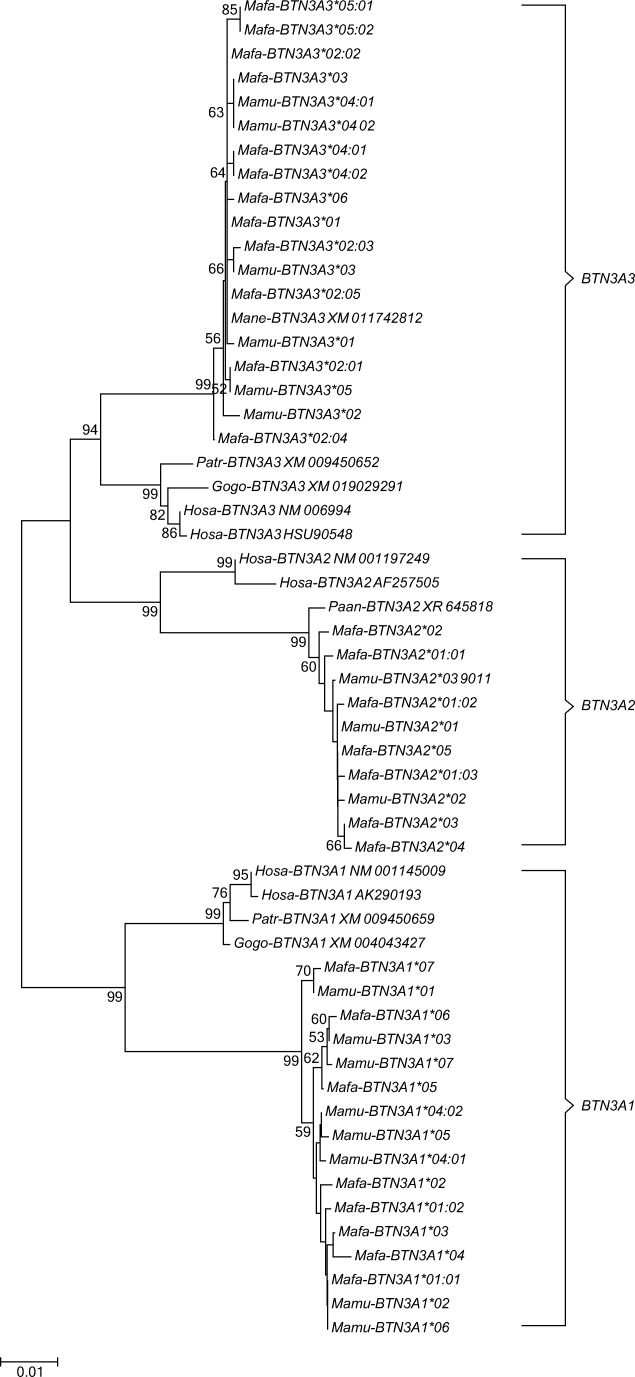
Phylogenetic tree of exons 2–6 of *BTN3A1*, *BTN3A2* and *BTN3A3* of different primate species. Sequences of the *BTN3A* genes defined in the two rhesus macaque and the two cynomolgus families have been compared to *BTN3A* sequences of humans (*Hosa*) and various NHP species as deposited in the NCBI database. *Gogo Gorilla gorilla*, *Patr Pan troglodytes*, *Mamu Macaca mulatta*, *Mafa Macaca fascicularis*, *Mane Macaca nemestrina*

Exons 7–9 of *BTN3A* genes have been excluded for phylogenetic analysis, since they are not comparable for all three *BTN3A* genes. The analysis of exons 2–6 showed that the alleles defined in this study cluster together with alleles of the same *BTN3A* genes of all other species analysed including hominoids (Fig. 5). This result confirms the observation that macaque *BTN3A* genes are true orthologs of their human equivalents (Afrache et al. 2017). In addition, the short branch lengths in the phylogenetic tree indicate that variation of the *BTN3A* genes is very low with regard to the number of variable nucleotides per allele. *BTN3A2* is the most conserved entity, as reflected by the lowest nucleotide and amino acid variation (Fig. 3a–c).

Furthermore, the positions of the variable, macaque-specific amino acids of all three BTN3A gene products are scattered over the coding sequence and not concentrated on certain positions (Fig. 3a–c), as contrasts the situation for the peptide binding site of the polymorphic MHC molecules (Hughes and Nei 1988). Exon 2 of *BTN3A1*, however, which encodes the IgV domain, forms an exception; the same four variable, macaque-specific amino acid positions are encoded within this exon, resulting in two different amino acids (Fig. 3a).

### *Mamu-* and *Mafa-BTN3A haplotype definition*

**Fig. 6:**
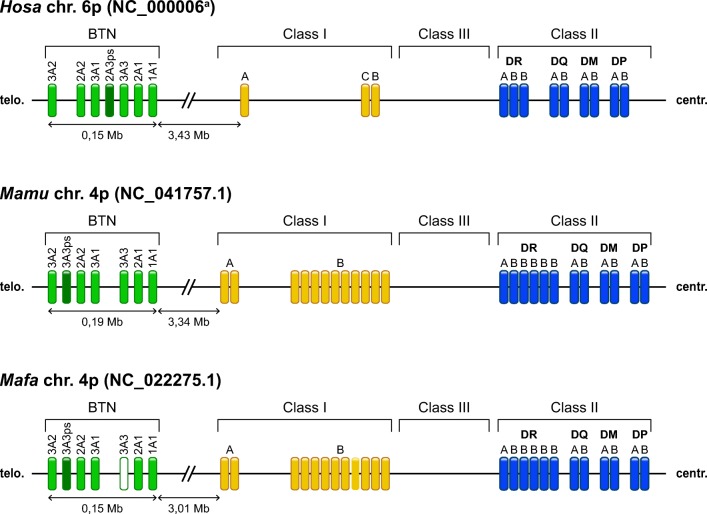
Schematic illustration of the localization of the *BTN1/2/3A* genes of humans, rhesus and cynomolgus macaques on the extended MHC class I region. The pseudogenes *Hosa-BTN2A3ps* and *Mamu/Mafa-BTN3A3ps* are marked by dark green squares. Although clearly defined in our experiments, *Mafa-BTN3A3* was not detected on the whole genome sequence (NC_022275.1). Therefore, its possible localization is indicated by an unfilled square. ^a^Horton et al. (2004)

Mapping of the various *Mamu-* and *Mafa-BTN* genes against published rhesus and cynomolgus whole genome sequences, respectively, showed the positions of the different *Mamu-* and *Mafa-BTN1/2/3A* genes on the extended MHC (https://www.ensembl.org/Macaca_mulatta/Info;https://www.ensembl.org/Macaca_fascicularis/Info/), which are highly comparable to the positions of the human *BTN* genes (Horton et al. 2004). (Fig. 6).

The co-segregation of the alleles of all three *BTN3A* genes and those of the *BTN3A3Like* pseudogene within the macaque families confirm duplication of the *BTN3A* genes and their localization in close proximity to each other on the chromosome. We were able to define 15 and 14 *BTN3A* haplotypes in the two rhesus and cynomolgus macaque families, respectively. Figure 2 and Suppl. Fig. 1 show the pedigrees of the two rhesus macaque families with their deduced *BTN3A* haplotypes, whereas in Fig. 4, the pedigrees of both cynomolgus macaque families and deduced *BTN3A* haplotypes are illustrated. Whereas the two rhesus macaque families share about half of their haplotypes, the two cynomolgus macaque families have no *BTN3A* haplotypes in common.

Additionally, mapping of the *BTN3A* genes within the extended MHC complex of the rhesus macaque is confirmed by the co-segregation of most of the *BTN3A* haplotypes with the *MHC* haplotypes defined beforehand. The co-segregation of *MHC* haplotypes and *BTN3A* alleles is also observed in the two cynomolgus macaque families (data not shown). As expected in accordance with the long distance between both regions (> 3 Mb), crossing over events between *BTN3A* and *MHC* haplotypes can be postulated as well as point mutations, leading to new allele/haplotype definitions. The deduced *MHC-BTN3A* haplotypes of the rhesus macaque family 2 are given as examples (Suppl. Fig. 1).

### AS of BTN3A in macaque species

**Fig. 7:**
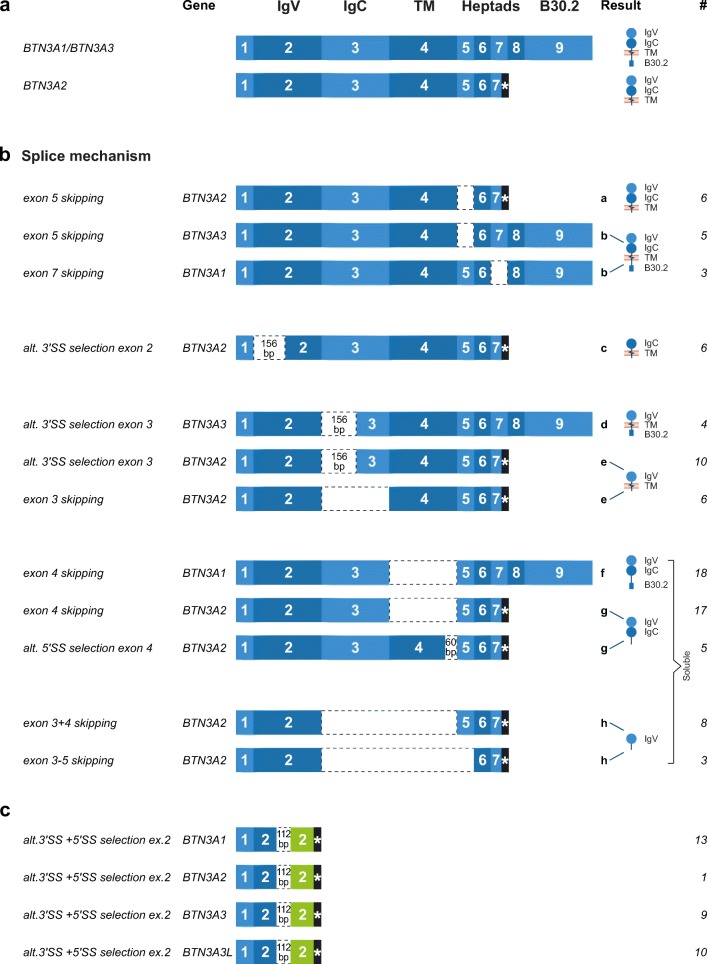
Schematic illustration of the *BTN3A* gene sequences, their resulting transcripts and proteins. **a***BTN3A1*, *BTN3A3* and *BTN3A2* genes, transcripts and predicted proteins using actual splice sites. **b** Various alternative splicing (AS) transcripts of *BTN3A* genes as observed in rhesus macaque family C68. Transcripts are categorized by the AS mechanism. These AS transcripts do not possess a premature stop codon and should therefore lead to a shorter but intact protein. **c** Illustration of one specific AS event occurring in all *BTN3A* genes and the pseudogene transcripts. This AS involves alternative 3′ splice site (SS) and 5′ SS selection within exon 2, which creates a 112-bp deletion in the middle of exon 2 and a frame shift (indicated in green) leading to a stop codon at the end of exon 2 (asterisk). Therefore, no gene product is expected

For many gene systems, high numbers of AS products showed up by way of the introduction of long-range next-generation sequencing (NGS) as PacBio Single Molecule Real-Time (SMART) sequencing compared to Sanger sequencing. By using Sanger sequencing of the different *BTN3A* cDNAs, however, an unexpectedly high number of various AS transcripts were observed in both macaque species in addition to the constitutive transcripts. AS products of BTN3A of nine rhesus macaques belonging to family 1 (Fig. 2) were analysed in detail. AS events, which have been observed more than three times, were categorized according to their splicing events as reviewed by Ast (2004) and Keren and co-workers (Keren et al. 2010). Figure 7 provides a summary of the different AS events, which may generate a shorter but in-frame transcript. We have observed exon skipping not only of exons 3, 4, 5 and 7 but also of multiple exons together (exons 3 + 4 and exons 3 to 5). Additionally, splice events mediated by alternative splice sites (SS), alternative 3′ SS and alternative 5′ SS were frequently observed, whereas in-frame splice events mediated by intron retention and cryptic exon inclusion were not noted. The alternatively spliced transcripts potentially result in eight different isoforms (Fig. 7b (a–h)): There are molecules that have an IgV and an IgC domain but no intracellular B30.2 part (Fig. 7b (a)); molecules with IgV, IgC and a B30.2 part but a heptad missing (Fig. 7b (b)); and molecules with only the IgC (Fig. 7b (c)) or the IgV (Fig. 7b (d, e)) domain. Additionally, AS transcripts are observed without the exons encoding the transmembrane part (Fig. 7b (f–h)), which could therefore result in soluble BTN3A isoforms. None of the observed splice events resulted in a soluble IgC domain. Exon skipping is the most frequently observed splicing mechanism that resulted in in-frame transcripts, as previously described for eukaryotes in general (Keren et al. 2010). Of all exon-skipping events, exon 4 skipping is the most frequent in *BTN3A1* (#18) and *BTN3A2* (#17) but has not been detected for *BTN3A3*. To substantiate the observed splice event and its absence in BTN3A3, we predicted and scored the alternative splice sites and compared them with each other using three different prediction models: namely, MaxEntScan (MES), Human Splice Finder (HSF) and Position Weight Matrix (PWM). These models did substantiate the observed skipping of exon 4 in transcripts of *BTN3A1* and *BTN3A2*, but the splicing strength scores could not explain the absence of this splice event in *BTN3A3* transcripts (Suppl. Table 2).

Nearly as often, splice events mediated by alternative 3′ SS (#6, #4, #10) were detected, whereas splice events mediated by alternative 5′ SS (#5) were far less frequently observed (Fig. 7b). Additionally, alternative splice sites resulted in the partial retention of introns, often in combination with exon skipping, but always introducing a stop codon so that no intact gene product would be formed. One of these splice events resulted in a deletion of 112 bp, and was mediated by alternative 3′ and 5′ splice sites in exon 2. This AS was frequently observed in all three *BTN3A* transcripts and the *BTN3ALike* pseudogene (Fig. 7c), although no *bona fide* gene product could be expected. All three in silico models predicted identical scores for the alternative splice sites that mediate the event in the transcripts of *BTN3A* (pseudo)genes. The PWM model did not detect the alternative 3′ SS at all. Compared to the actual splice site scores, the alternative splice site scores were lower for all models, suggesting that constitutive splicing is dominant over this alternative splicing event (Suppl. Table 2).

## Discussion

The distribution analysis of *BTN3A* genes in rhesus and cynomolgus macaque families confirmed the presence of the three genes *BTN3A1*, *BTN3A*2 and *BTN3A3* in all animals tested (Afrache et al. 2017). In addition, the discovery of a pseudogene, named *BTN3A3Like*, was facilitated by exon 2 analysis. This pseudogene appears, however, to be absent in hominoids, who instead have a *BTN2A* pseudogene (Rhodes et al. 2016). This observation confirms the flexibility of the region not only in humans but also in OWM such as macaques.

Allelic variation of all *BTN3A* genes was documented for rhesus as well as cynomolgus macaques, although the degree of polymorphism is quite low, with the lowest variation observed for *BTN3A2* followed by *BTN3A3* and *BTN3A1.* The latter gene has, however, a remarkably conserved exon 2 (IgV region), and no non-synonymous mutations in exon 4 (TM) and exons 6–8 (heptads) were observed. *BTN3A3* shows no non-synonymous variations in exons 5–8 (heptads) and only one in exon 4 (TM region) and exon 9 (intracellular B30.2 part), respectively. As may be expected for a pseudogene, which experiences no purifying selection, the allelic variation observed in exon 2 of *BTN3A3Like* is higher than in those of the *BTN3A* genes (Fig. 2). In concordance with the level of polymorphism observed in macaque MHC genes (Doxiadis et al. 2013), *BTN3A* alleles and haplotypes are more variable in cynomolgus monkeys than in rhesus macaques of Indian origin as represented in our cohort.

Furthermore, phylogenetic analysis of exon 2 showed that HOM exon 2 sequences of the three *BTN3A* genes fall apart from the gene clusters of OWM and that they do not branch in a gene-specific manner as do the alleles of OWM (Fig. 1). The homogenization, replacing IgV-encoding sequences of *BTN3A1*, *BTN3A2* and *BTN3A3* in HOM, has recently been described (Afrache et al. 2017). This phenomenon appears to be the result of an ancient recombination between the 5′ region of a *BTN3A1Like* gene and the 3′ region of a *BTN3A2-like* gene, resulting in a chimeric *BTN3A3* gene. Our data suggest that this ancient recombination process took place after the divergence of OWM and HOM ~ 25 myr ago, since the homogenization is not observed in OWM.

In addition to the exon 2 sequences of *BTN3A3Like* genes of diverse macaque species (Fig. 1), a database search revealed the presence of a *BTN3A3Like* sequence in the olive baboon. In contrast to the macaque pseudogene sequences, however, which all have the stop codon at amino acid 94 in exon 2, the olive baboon sequence already has a stop codon at amino acid 32. In the latter, a sequence of 258 bp is integrated after exon 1, which seems to originate from the 3′ untranslated region of the *BTN3A2* gene*.* The existence of such a chimeric pseudogene seems to provide additional evidence that intragenic recombination processes are a common feature within the BTN region.

In contrast to the phylogenetic analysis of exon 2, the phylogeny of the *BTN3A* transcripts of exons 2–6 of human and non-human primate sequences shows a gene-specific clustering (Fig. 5), confirming that macaque *BTN3A* genes are true orthologs of their human equivalents.

Based on the relationship of the animals and segregation profiles within the rhesus and cynomolgus macaque families, we were able to define several *BTN3A* haplotypes. In concordance with the higher polymorphism observed in cynomolgus monkeys, haplotypes are more variable in this species than they are in rhesus macaques, most probably due to their various origins from different parts of Indonesia. Physical mapping showed the localization of the different macaque *BTN* genes within the extended MHC class I region, which is comparable to the position of the human BTN region (Fig. 6). Frequent co-segregation of *BTN3A* alleles/haplotypes with the MHC haplotypes verifies their localization as has been described for humans (Horton et al. 2004).

Already at the beginning of the century, AS was observed by Sanger sequencing for human BTN3A transcripts, indicating that AS occurs far more frequently in these genes than in others (Rhodes et al. 2001). Recently, in BG genes, which represent family members of the BTN gene family in chicken, AS has also been defined by Sanger sequencing (Chen et al. 2018). A remarkable phenomenon is the high degree of AS that has been observed for alleles of all *BTN3A* genes and the pseudogene. In general—e.g. for MHC class I and II genes—AS is rarely revealed using the low-resolution Sanger sequencing technique and is only detected when NGS techniques have been used and even then in relatively low numbers. The fact that AS isoforms are detected by the Sanger method for macaque BTN3A genes suggests that these transcripts are generated at a fairly high frequency. One could imagine that isoforms of the BTN3A molecules that have been generated due to AS may play a specific role as Vγ9Vδ2 T cell modulators. Exon skipping comprised the most frequently observed AS events, especially those that included exon 4, which encodes for the transmembrane part section, as was observed for *BTN3A1* and *BTN3A2*. Predicting the actual splice sites with three different in silico models did not answer the question as to why exon 4 skipping does not occur in *BTN3A3*. Intron 3, which is situated next to exon 4, does show a high degree of nucleotide variation between the different *BTN3A* genes. Moreover, intron 3 of *BTN3A3* is longer than the equivalent structure of the other two *BTN3A* genes. All these differences may influence alternative splicing—for example, through the existence of additional silencer/enhancer motives and/or the formation of secondary structures, which may prevent the appearance of a certain exon within the transcript. It is therefore plausible to suggest that the intron sequences may be the reason that this AS event can be detected in the transcripts of only two of the three *BTN3A* genes.

Exon skipping that includes exon 4, which encodes the transmembrane section, may result in the generation of soluble molecules. Gu et al. (2018) have presented a model that postulates that BTN3A isoforms help BTN3A1, the pAg-sensing molecule expressed on the cell surface, to make a conformational change when activated. This change, including possible multimerization events, can then be recognized by Vγ9Vδ2 T cell receptors. Soluble molecules may be especially supportive in this process. In this case, the frequently observed AS isoforms may actually represent a regulation phenomenon that helps in activating BTN3A-positive cells. We mainly looked at transcripts of white blood cells, which are a proxy for what may happen elsewhere in the body. One wonders of course whether the AS profiles would be different in tissues in healthy or stressed conditions. Indeed, soluble BTN3A isoforms are detected in the supernatant of pancreatic cell lines and the plasma of pancreatic ductal adenocarcinoma patients. Soluble BTN3A and BTN3A1 were associated with a decreased overall survival rate of these patients (Benyamine et al. 2016; Blazquez et al. 2018). Consequently, soluble BTN3A molecules can serve as prognostic biomarkers, highlighting the value of macaques in clinical cancer research, in addition to their importance in future proof-of-concept studies for the protective features of Vγ9Vδ2 T cells—for instance, against *M. tuberculosis* or other infectious agents (Chen 2016).

## Electronic supplementary material

Suppl. Fig. 1. Pedigree of rhesus macaque family EAW with deduced *BTN3A* and *MHC* haplotypes. *Blue squares* represent male and *red ovals* female animals. *Grey squares/ovals* indicate the animals that have not been typed for BTN3A. *Letters* (a-o) indicate the *BTN3A* haplotype defined in this family. Below the pedigree, the *BTN3A* haplotypes (a-o) are listed. The subdivision in a1 or a2 and in c1-c4 indicates that a certain *BTN3A* haplotype is associated with a different MHC haplotype. (PDF 333 kb)

Suppl. Table 1 (DOCX 18 kb)

Suppl. Table 2 (XLSX 11 kb)
