# Supplementary material for: Analysis of macaque BTN3A genes and transcripts in the extended MHC: conserved orthologs of human γδ T cell modulators
Source: Immunogenetics. 2019 Aug 5;71(8):545–59. doi: 10.1007/s00251-019-01126-9 (PMC6790196; doi:10.1007/s00251-019-01126-9)
Supplement: Supplementary file 1 — Pedigree of rhesus macaque family EAW with deduced BTN3A and MHC haplotypes. Blue squares represent male and red ovals female animals. Grey squares/ovals indicate the animals that have not been typed for BTN3A. Letters (a-o) indicate the BTN3A haplotype defined in this family. Below the pedigree, the BTN3A haplotypes (a-o) are listed. The subdivision in a1 or a2 and in c1-c4 indicates that a certain BTN3A haplotype is associated with a different MHC haplotype. (PDF 333 kb) [file 251_2019_1126_MOESM1_ESM.pdf]

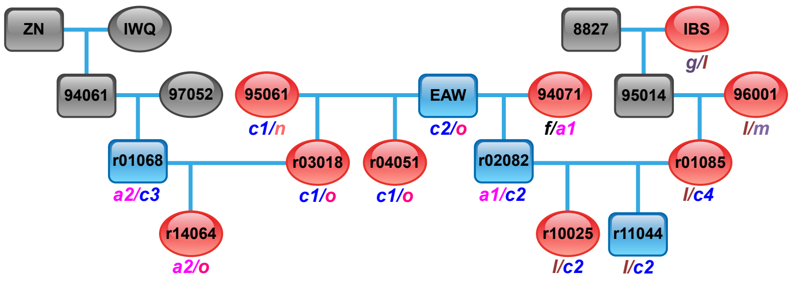

	BTN3A1	*BTN3A2*	*BTN3A3*	MHC-A	MHC-B	MHC-DRB
a1	*BTN3A1*01*	*BTN3A2*01*	*BTN3A3*01*	A004	B024a	DRB04
a2	*BTN3A1*01*	*BTN3A2*01*	*BTN3A3*01*	A004	B012c	DRB03f
c1	*BTN3A1*05*	*BTN3A2*01*	*BTN3A3*01*	A002a	B001a	DRB02
c2	*BTN3A1*05*	*BTN3A2*01*	*BTN3A3*01*	A004	B015a	DRB04
c3	*BTN3A1*05*	*BTN3A2*01*	*BTN3A3*01*	A001	B048	DRB03a
c4	*BTN3A1*05*	*BTN3A2*01*	*BTN3A3*01*	A023	B012b	DRB03f
g	*BTN3A1*04:02*	*BTN3A2*01*	*BTN3A3*02*	A012	B008	DRB10
l	*BTN3A1*02*	*BTN3A2*01*	*BTN3A3*04:01*	A006	B069a	DRB13a
m	*BTN3A1*06*	*BTN3A2*01*	*BTN3A3*04:02*	A026	B047a	DRB03e
n	*BTN3A1*04:02*	*BTN3A2*01*	*BTN3A3*05*	A016	B024a	DRB04
o	*BTN3A1*04:01*	*BTN3A2*01*	*BTN3A3*02*	A002a	B043a	DRB01a
