# Supplementary material for: Analysis of macaque BTN3A genes and transcripts in the extended MHC: conserved orthologs of human γδ T cell modulators
Source: Immunogenetics. 2019 Aug 5;71(8):545–59. doi: 10.1007/s00251-019-01126-9 (PMC6790196; doi:10.1007/s00251-019-01126-9)
Supplement: Supplementary file 2 — (DOCX 18 kb) [file 251_2019_1126_MOESM2_ESM.docx]

Supplementary Table1: BTN3A primers

| Primer Name | Sequence 5’- 3’ | Product Size (Bp) |
| --- | --- | --- |
| BTN3A1_IgV_For | TGT CTG AGA AGC ACC CTT CC | 531 |
| BTN3A1_IgV_Rev | ATT GCT AGT GAG CAG CCA GG |  |
| BTN3A2_IgV_For | TTC CCT GTC TGA GAA GGA CC | 536 |
| BTN3A2_IgV_Rev | ATT GCT AGT GAG CAG CCA GG |  |
| BTN3A3_IgV_For | TTC CCT GTC TGA GAA GGA CC | 536 |
| BTN3A3_IgV_Rev | ATT GCT AGT GAG CAG CCA GG |  |
|  |  |  |
| Mamu/Mafa-BTN3A1_Fex1sp | CCT TTT GCT TCA GCT GCT CAT G | 1528 |
| Mamu/Mafa-BTN3A1_R2sp | CAC CAC TCG GTC AGA ATT GGA |  |
| Mamu/Mafa-BTN3A2_Fex1sp | TCT TGG TCC AGC TGC TCA CT | 1770 |
| Mamu/Mafa-BTN3A2_R2sp | ATC TCT CAG GGT TGT CCG GT |  |
| Mamu/Mafa-BTN3A3_Fex1sp | ACA GAC CTT TTT GGC AGA GCA C | 1676 |
| Mamu/Mafa-BTN3A3_R1sp | GCA GGG AGA AGC AGA GAT GTT G |  |
| Mamu-BTN3A3Like_Fex2sp | TTT GCT GTG CTT GGA CCC CT | 711 |
| Mamu-BTN3A3Like_Rex4sp | AAC TGG CTC CTG CAA GAA GC |  |
